# Supplementary material for: Nonclassical Monocytes Are Prone to Migrate Into Tumor in Diffuse Large B-Cell Lymphoma
Source: Front Immunol. 2021 Dec 16;12:755623. doi: 10.3389/fimmu.2021.755623 (PMC8716558; doi:10.3389/fimmu.2021.755623)
Supplement: Supplementary file 1 [file DataSheet_1.docx]

**Supplemental figure legends**

**Figure S1: Gating strategy**

**Figure S2 (related to Figure 1): All monocyte subsets are increased in B-cell lymphomas**

A- Monocyte subsets counts in peripheral blood from HD (n = 6), follicular lymphomas (n = 9), mantle cell lymphomas (MCL, n = 9), chronic lymphocytic leukemias (CLL, n = 11), and marginal zone lymphomas (SMZL, n = 10). On these patients, flow cytometry was performed on cryopreserved cells. B- Ratio between MO (classical and intermediate) and ncMO in B-cell lymphomas.

**Figure S3 (related to Figure 2): Q-PCR analysis, unsupervised hierarchical clustering for cMO and iMO from DLBCL and HD patients**

cMO and iMO were sorted from 7 DLBCL and 4 HD before analysis by high-throughput Q-PCR for genes listed in Table S3. Pearson's correlation and complete linkage were employed.

**Figure S4 (related to Figure 3): Q-PCR analysis, unsupervised hierarchical clustering for ncMO Slan^pos^ and ncMO Slan^neg^ from DLBCL and HD patients**

ncMO Slan^pos^ and ncMO Slan^neg^ were sorted from 7 DLBCL and 4 HD before analysis by high-throughput Q-PCR for genes listed in Table S3. Pearson's correlation and complete linkage were employed.

**Figure S5 (related to Figure 3): Q-PCR analysis, unsupervised hierarchical clustering for cMO, iMO, ncMO Slan^pos^ and ncMO Slan^neg^ from DLBCL patients**

cMO, iMO, ncMO Slan^pos^, and ncMO Slan^neg^ were sorted from 7 DLBCL before analysis by high-throughput Q-PCR for genes listed in Table S3. Pearson's correlation and complete linkage were employed.

**Figure S6 (related to Figure 3): Q-PCR analysis, unsupervised hierarchical clustering for cMO, iMO, ncMO Slan^pos^ and ncMO Slan^neg^ from DLBCL and HD patients**

cMO, iMO, ncMO Slan^pos^, and ncMO Slan^neg^ were sorted from 7 DLBCL and 4 HD before analysis by high-throughput Q-PCR for genes listed in Table S3. Pearson's correlation and complete linkage were employed.

**Figure S7 (related to Figure 5): High levels of circulating ncMO is an adverse event in DLBCL.**

Event-free survival (EFS) in training cohorts (NCT01287923) Threshold of the ratio of ncMO to other monocytes parameter was defined on the training cohort using the maxstat package.

**Table S1: Antibodies for fluorescent flow cytometry analysis**

| **Target** | **Fluorochrome** |  | **Clone** | **Company** |
| --- | --- | --- | --- | --- |
| HLA-DR | PE-CF594 |  | G46-6 | BD Biosciences |
| CD14 | PC7 |  | RMO52 | Beckman Coulter |
| CD16 | APC-Alexa700 |  | 3G8 | Beckman Coulter |
| CD3 | V450 |  | UCHT1 | BD Biosciences |
| CD335 | BV421 |  | 9E2/NKp46 | Biolegend |
| CD45 | Krome Orange |  | J33 | Beckman Coulter |
| Slan | FITC |  | DD-1 | Miltenyi Biotec |

**Table S2: Antibodies or parameters used for mass cytometry analysis**

| **Target /**  **Compound** | **Metal /**  **Parameter** |  | **Clone** | **Company** | **Staining** |
| --- | --- | --- | --- | --- | --- |
| CD11b | 141Pr |  | ICRF44 | Biolegend | C |
| CD19 | 142Nd |  | HIB19 | Fluidigm | - |
| CD366 Tim3 | 143Nd |  | F38-2E2 | Biolegend | C |
| Slan-FITC / anti-FITC | 144Nd |  | DD-1 | Miltenyi Biotech / Fluidigm | I |
| MerTK-PE / anti-PE | 145Nd |  | 125518 | R&D systems / Fluidigm | I |
| CD64 | 146Nd |  | 10.1 | Fluidigm | - |
| CD36 | 147Sm |  | 5-271 | Biolegend | C |
| CD164 | 148Nd |  | 67D2 | Biolegend | C |
| CCR2 | 149Sm |  | K036C2 | Biolegend | C |
| CD43 | 150Nd |  | 84-3C1 | Fluidigm | - |
| CD123 | 151Eu |  | 6H6 | Fluidigm | - |
| CD13 | 152Sm |  | WM15 | Fluidigm | - |
| CD45RA | 153Eu |  | HI100 | Fluidigm | - |
| CD163 | 154Sm |  | GHI/61 | Fluidigm | - |
| CD27 | 155Gd |  | L128 | Fluidigm | - |
| CD86 | 156Gd |  | IT2.2 | Fluidigm | - |
| CD33 | 158Gd |  | WM53 | Fluidigm | - |
| CD11c | 159Tb |  | Bu15 | Fluidigm | - |
| CD14 | 160Gd |  | M5E2 | Fluidigm | - |
| CD32 | 161Dy |  | FUN-2 | Biolegend | C |
| S100A9-APC / anti-APC | 162Dy |  | MRP-14 | Biolegend / Fluidigm | I/intra |
| HLA-DR | 163Dy |  | L243 | Biolegend | C |
| CD206 | 164Dy |  | 3.29B1.10 | Beckman Coulter | C |
| CD16 | 165Ho |  | 3G8 | Fluidigm | - |
| CD120a | 166Er |  | 80M2 | Beckman Coulter | C |
| CCR7 | 167Er |  | G043H7 | Fluidigm | - |
| CD8 | 168Er |  | SK1 | Fluidigm | - |
| CD25 | 169Tm |  | 2A3 | Fluidigm | - |
| CD3 | 170Er |  | SP34-2 | Fluidigm | - |
| CD68 | 171Yb |  | Y1/82A | Fluidigm | Intra |
| CD9 | 172Yb |  | SN4 C3-3A2 | Fluidigm | - |
| CD45 | 173Yb |  | 2D1 | Biolegend | C |
| CD279 | 174Yb |  | EH12.2H7 | Biolegend | C |
| CD274 | 175Yb |  | 29E.2A3 | Fluidigm | - |
| CD127 | 176Yb |  | A019D5 | Fluidigm | - |
| Iridium | 191Ir |  | - | Fluidigm | - |
| Iridium | 193Ir |  | - | Fluidigm | - |
| Cisplatin | 195Pt |  | - | Enzo Life Sciences | - |
| - | Cell length |  | - | - | - |

Staining: C: Custom conjugate; I: Indirect staining; intra: intrastaining

**Table S3: List of genes evaluated by high-throughput Q-PCR on monocyte subsets**

| *ADAM17* | *CD64* | *IL12A* | *RELB* |
| --- | --- | --- | --- |
| *AIM2* | *CD68* | *IL17R* | *S100A12* |
| *BclxL* | *CD80* | *IL6* | *S100A8* |
| *CEBPb* | *CD86* | *IL6R* | *S100A9* |
| *C5aR* | *CTLA4* | *LAG3* | *SLC7A11* |
| *caspase1* | *CXCL1* | *MMP9* | *STAT1* |
| *CCR2* | *CXCL10* | *MYD88* | *STAT3* |
| *CCR5* | *CyclinD1* | *NFKBP50* | *STAT6* |
| *CD11b* | *EP24* | *NFKBP52* | *TGFb* |
| *CD137* | *GCSFR* | *NIK* | *TGM2* |
| *CD14* | *Galectin9* | *NLRC4* | *Tim3* |
| *CD16* | *GMCSFR* | *NOX2* | *TLR2* |
| *CD163* | *HIFa* | *OX40* | *TLR4* |
| *CD32* | *HLADR* | *PD1* | *TLR6* |
| *CD33* | *HO1* | *PDL1* | *TNFa* |
| *CD36* | *IDO* | *PDL2* | *TNFAIP6* |
| *CD38* | *IL4R* | *RAGE* | *TNFR2* |
| *CD40* | *IL10* | *RELA* |  |

**Table S4: QPCR data**
