# Supplementary material for: Nonclassical Monocytes Are Prone to Migrate Into Tumor in Diffuse Large B-Cell Lymphoma
Source: Front Immunol. 2021 Dec 16;12:755623. doi: 10.3389/fimmu.2021.755623 (PMC8716558; doi:10.3389/fimmu.2021.755623)
Supplement: Figure S1 — Gating strategy. [file Image_1.pdf]

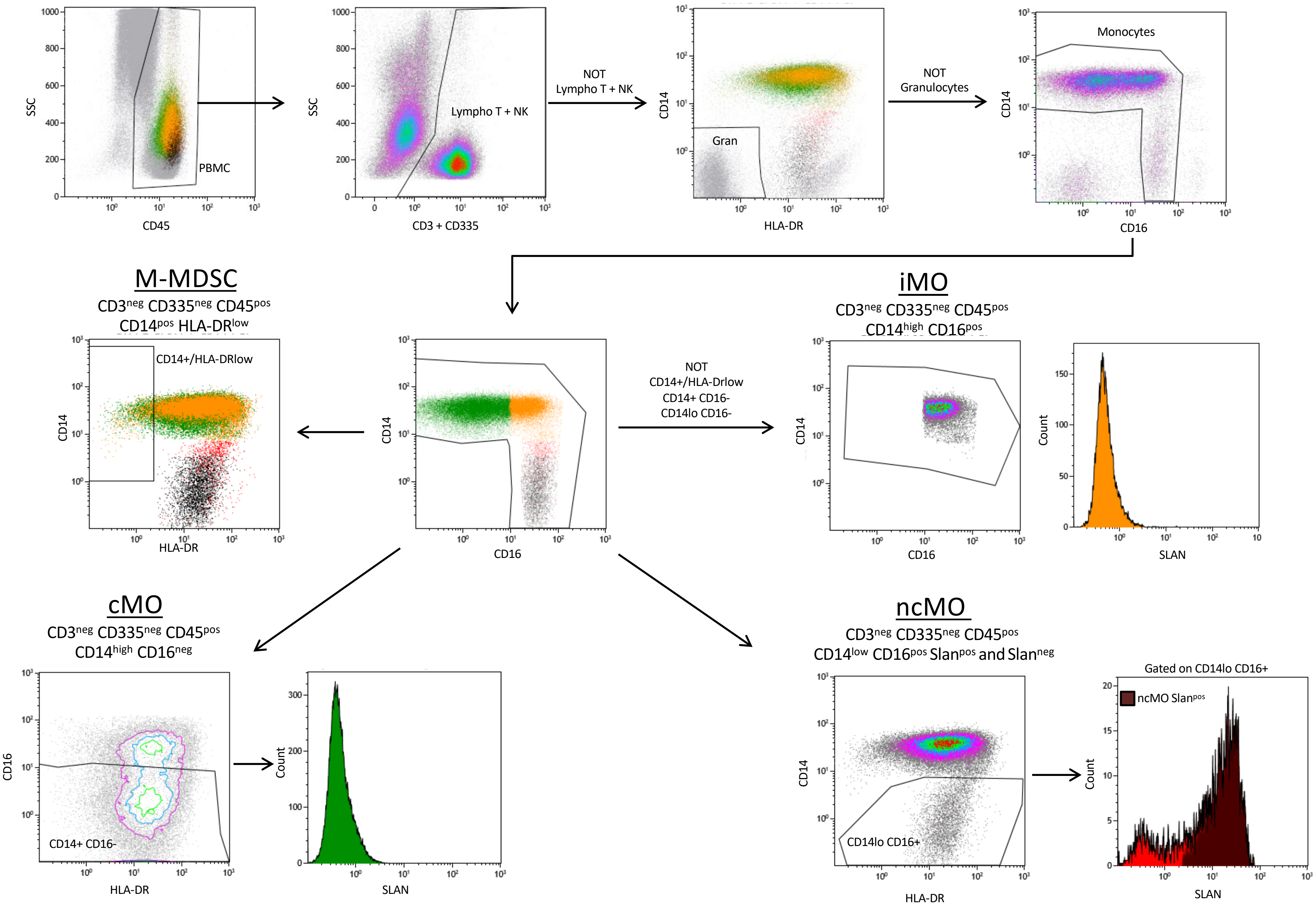

SSC
CD45
PBMC
CD3 + CD335
Lympho T + NK
NOT
Lympho T + NK
CD14
HLA-DR
Gran
NOT
Granulocytes
Monocytes
CD16
M-MDSC
CD3neg CD335neg CD45pos
CD14pos HLA-DRlow
CD14+/HLA-DRlow
NOT
CD14+/HLA-Drlow
CD14+ CD16-
CD14lo CD16-
iMO
CD3neg CD335neg CD45pos
CD14high CD16pos
Count
SLAN
cMO
CD3neg CD335neg CD45pos
CD14high CD16neg
CD14+ CD16-
ncMO
CD3neg CD335neg CD45pos
CD14low CD16pos Slanpos and Slanneg
CD14lo CD16+
Gated on CD14lo CD16+
ncMO Slanpos
